# 

**Published:** 1892-04

**Authors:** 


### CONTENTS

ORIGINAL COMMUNICATIONS—
The Pathology and Treatment of Pneumonia. By J. Clarence Johnson, M. D., Atlanta, Ga. 65
Peculiar Idiosyncrasy—Treatment Wanted. By J. L. Bass, M. D., Brewton, Ala. 74
TRANSLATION—
Concerning Value of Partial Operations in the Treatment of Cancer of the Cervix. By Dr. E. Monod, Bordeaux, France. 75
SOCIETY REPORTS—
Clinical Society of Maryland 80
The Atlanta Society of Medicine 87
CORRESPONDENCE—
New York Letter 91
EDITORIAL—
The Change in the Journal 97
The Treatment of Tuberculosis by Guaiacol 98
Treatment of Anæmia by Arsenite of Copper 100
The Bichloride of Gold Industry 101
The Medical Association 102
SELECTIONS 103-121
MEDICAL ITEMS 122-124
BOOK REVIEWS 125-128
INDEX TO ADVERTISEMENTS xiii
ITEMS OF INTEREST xi and xii
CLUB RATE PAGE xiv

